# Capture-recapture analysis of all-cause mortality data in Bohol, Philippines

**DOI:** 10.1186/1478-7954-9-9

**Published:** 2011-04-17

**Authors:** Karen L Carter, Gail Williams, Veronica Tallo, Diozele Sanvictores, Hazel Madera, Ian Riley

**Affiliations:** 1 School of Population Health, University of Queensland, Herston (Brisbane), Queensland, Australia; 2 Research Institute of Tropical Medicine, Manila, Philippines

## Abstract

**Background:**

Despite the importance of mortality data for effective planning and monitoring of health services, official reporting systems rarely capture every death. The completeness of death reporting and the subsequent effect on mortality estimates were examined in six municipalities of Bohol province in the Philippines using a system review and capture-recapture analysis.

**Methods:**

Reports of deaths were collected from records at local civil registration offices, health centers and hospitals, and parish churches. Records were reconciled using a specific set of matching criteria, and both a two-source and a three-source capture-recapture analysis was conducted. For the two-source analysis, civil registry and health data were combined due to dependence between these sources and analyzed against the church data.

**Results:**

Significant dependence between civil registration and health reporting systems was identified. There were 8,075 unique deaths recorded in the study area between 2002 and 2007. We found 5% to 10% of all deaths were not reported to any source, while government records captured only 77% of all deaths. Life expectancy at birth (averaged for 2002-2007) was estimated at 65.7 years and 73.0 years for males and females, respectively. This was one to two years lower than life expectancy estimated from reconciled reported deaths from all sources, and four to five years lower than life expectancy estimated from civil registration data alone. Reporting patterns varied by age and municipality, with childhood deaths more underreported than adult deaths. Infant mortality was underreported in civil registration data by 62%.

**Conclusions:**

Deaths are underreported in Bohol, with inconsistent reporting procedures contributing to this situation. Uncorrected mortality measures would subsequently be misleading if used for health planning and evaluation purposes. These findings highlight the importance of ensuring that official mortality estimates from the Philippines are derived from data that have been assessed for underreporting and corrected as necessary.

## Background

Effective planning and monitoring of health services requires accurate measures of mortality by age and sex. Additionally, population health targets such as the Millennium Development Goals [1] require reliable data to measure progress. Mortality data in the Philippines are collected through the civil registration system, the health system, and the parish records of local churches.

Despite the importance of mortality data, official reporting systems rarely capture every death [2]. The challenge for public health planners in many countries is to meaningfully interpret the available data. A global assessment of mortality data in 2003 listed the Philippines as having only "medium" quality death records, with reporting completeness estimated at 77% based on data supplied to the World Health Organization [2]. The completeness of death reporting and its impact on mortality estimates was examined in six municipalities of Bohol province in the Philippines using a system review and capture-recapture analysis. Variations in reporting patterns by age group, sex, and municipality were also examined.

Bohol is a province of the Central Visaya region of the Philippines. More than 95% of the provincial population and study area is Catholic [3], although there is a small but localized Muslim population near the provincial capital, Tagbilaran City. Major industries are agriculture and fishing [4]. The province has a population of approximately 1,227,809 (2007) [3] living in 47 municipalities and one city.

Capture-recapture analysis uses the number of deaths recorded by each combination of sources to estimate the number of unreported deaths, and therefore the estimated total number of deaths [5]. Capture-recapture methods have been used extensively to obtain estimates of disease incidence and to assess coverage of disease-specific surveillance systems and registers [6-10]. For all-cause mortality, this method provides an additional tool to quantify and correct underregistration of deaths. Other methods compare reported deaths to expected patterns based on age distribution or require repeated censuses to evaluate survival [11,12].

## Methods

The study included Baclayon, Balilihan, Cortes, Dauis, and Panglao municipalities, and Tagbilaran city. These were selected as part of a broader study to improve existing methods for estimating mortality levels from incomplete data and verbal autopsy methods for assigning cause of death under the Gates Grand Challenges in Global Health program [13]. The municipalities selected were included in this larger study due to their proximity and subsequent access to hospitals in Tagbilaran city.

### System review

A system review was conducted to assess data collection and management within municipal mortality reporting systems. The review comprised observation and mapping of processes; examination of forms, policy documents, and electronic systems; and key informant interviews held with local civil registrars, health officers and health center staff, hospital staff, church officials, cemetery managers, and staff of the provincial branch of the National Statistics Office (NSO).

### Data Collection

Project staff visited local civil registration offices, health centers and hospitals, and parish churches regularly during 2006-2007 and extracted death report data from log books and registers using a standard form. Key variables collected were: municipality of report, municipality of residence, name of agency, nature of agency (local civil registry [LCR], health center [HC], or church), full name, sex, date of birth, date and place of death, and age at death. Population by age group, sex, and municipality was derived from the 2000 [14] and 2007 [15] censuses using exponential interpolation to provide estimates for intermediate years.

### Data reconciliation

Each death was assigned a unique number. Records were reconciled to create a single list of unique deaths indicating source(s) in which each was recorded. Project staff manually matched deaths by comparing data extraction forms from all sources. At least three of five criteria had to be met for records to be considered a match: same full name (minor variations in spelling, variations in name order or a phonetic match were allowed), age at death within one year, date of death within five days, same municipality of residence, or same sex. If more than one record was found as a match, each record was required to share three criteria with another record for the individual. A large number of church records could not be matched because of incomplete entries, and it was consequently decided that outstanding records matching on full name and date of death would also be included. Records were then entered into an Access database [16] that was analyzed to confirm all matches met stated criteria and no matches had been missed, to remove duplicate records, and to correct minor data entry errors. Deaths with missing age for a particular source were redistributed according to two imputed age distributions: (a) for that source, and (b) for all sources combined. In cases where matched records did not have identical municipality or age at death, these variables were recorded separately, and sensitivity analyses were used to examine the influence of such mismatches.

The following measures of mortality were calculated using standard methods [12]: age-specific mortality rate; infant mortality rate (IMR) (probability of dying before 1 year of age); childhood mortality (probability of dying before 5 years of age); adult mortality (probability of dying between 15 and 59 years of age inclusive); and life expectancy at birth (LE).

### Two-source capture-recapture analysis

A key assumption in a two-source analysis is that sources are independent, i.e., reporting to one source does not influence the chance of the event being reported to the other source [17-19]. The system review revealed that LCR and HC were highly interdependent and so they were combined; this combination is henceforth referred to as the "official" source. Unreported deaths were estimated using a two-source capture-recapture analysis of official and church sources. Data were analyzed using Excel and SAS. Under the assumption that official and church sources are independent, the number of missing deaths (x) may be estimated as bc/a, where a, b, c are the numbers of deaths found by both sources (a), only in the official records (b), and only in the church records (c) [17]. Confidence intervals were calculated using the maximum likelihood estimator. Unreported deaths were estimated for total deaths, with subgroup analyses for sex, age, and municipality. The estimated percentage of underreporting was examined by subgroup to identify variation in reporting patterns. Dependence between official data and church data was assessed using the method of Hook and Regal [20], with a value of 1 signifying sources are independent, and a positive and negative dependency between sources indicated by values less than and greater than 1, respectively.

### Three-source capture-recapture analysis

Three-source capture-recapture analysis allows for pair-wise dependence between sources [6-8,17], and used LCR, HC, and church data separately. Data were analyzed using the SAS CATMOD procedure [21] for total deaths as well as by municipality and age group. Age was grouped as 0-4 years, 5-14 years, and 15+ years. Each three-source analysis resulted in eight possible models (Table 1). The minimal three-source model uses three parameters, one for each source, and assumes independence of all sources [7,17]. An extended model incorporates three additional parameters, which allows the estimation of the three possible pair-wise dependencies (LCR and HC, LCR and church, and HC and church), generating three models with a single pair-wise dependency combination, and three models incorporating two pair-wise dependencies. The "saturated model" has an additional parameter for modification of each pair-wise dependence by the third source [7,17]. Models were selected on the basis of the system evaluation (qualitative assessment) and statistical criteria such as the significance of associations between sources (using likelihood ratio tests) and goodness-of-fit criteria (Bayesian Information Criterion (BIC) and the Akaike Information Criterion (AIC)) [17]. Excel and STATA were used for analysis [22].

**Table 1:** Models from three-source capture-recapture analysis (all ages) for Bohol study area, 2002-2007

Model*	d.f	G2	p-value	AIC	BIC	Estimator (x)	Total (N)	Lower 95% CI	Upper 95% CI
Independent	3	1900.00	<0.0001	1894	1879	147	8222	8208	8236

1-2	2	77.43	<0.0001	73	63	382	8457	8422	8493

1-3	2	1800.00	<0.0001	1796	1786	69	8144	8130	8157

2-3	2	1900.00	<0.0001	1896	1886	148	8223	8206	8240

1-2,1-3	1	69.75	<0.0001	68	63	520	8595	8476	8715

1-2, 2-3	1	16.50	<0.0001	15	9	552	8627	8560	8695

1-3, 2-3	1	1800.00	<0.0001	1798	1793	62	8137	8123	8150

1-2, 1-3, 2-3	0	-0.0002	1.000	0	0	908	8983	8748	9218

Source 1 - LCR data; Source 2 - health data; Source 3 - church data

* Notation indicates associations between sources taken into account by the model, i.e., the 1-2 model accounts only for an association between source 1 (LCR) and source 2 (health center)

### Final mortality estimates

Final estimates of the total number of deaths were derived from both the two- and three-source analyses. Models derived from each of these were assessed for plausibility and fit based on the statistical techniques described above and the system assessment. Measures of mortality, including IMR, childhood mortality, LE, and adult mortality, were then recalculated using the corrected estimates.

## Results

### System Analysis

Filipino legislation [23,24] requires families of the deceased person, regardless of the cause of death, to obtain a medical certificate of death and to present this to the Local Civil Registrar to obtain an official death certificate prior to burial. Nearly all funerals are overseen by the local Catholic Church, which also keeps a record of deaths. Stillbirths are recorded using separate forms and do not appear on the death registers.

At the local registry office, the death is recorded in the register book and a death certificate issued to the family. A copy of the death certificate is sent to the National Statistics Office along with an electronic file if the municipality also maintains an electronic database. Health records may be derived from clinic log books, hospital separation data, or the medical certificate of death. While any medical practitioner can issue a certificate of death, Filipino law requires that each medical certificate of death is submitted to the local municipal health officer for review and signature [24]. A record of the death should be available from the health center even if the medical certificate was not originally issued there. Church records include the name of the deceased, date of death, age at death, usual place of residence, and place of death. These records are maintained in a standardized format as set out in log books sold to the parishes through the church hierarchy.

The system review demonstrated significant dependence in the reporting of deaths between the LCR and the HC. Families often approached both health and civil registry offices independently, but medical certificates of death were also forwarded directly by several municipal health offices to the LCR. HCs either recorded deaths directly from the medical certificate of death at the time it was issued or reviewed, or else kept no record of the death at that time and later relied on either a monthly report of deaths from the LCR or on the family returning a copy of the official death certificate to the health office after registration. There was no official contact between the churches and government offices and no apparent dependence in reporting practices.

In practice, there was considerable variation in reporting processes across the six municipalities. Noted variations included whether families were required to first report the information to the local registrar or health center, whether there was someone present at the health center who could certify cause of death, and the charges associated with reporting. Practices for dealing with the nonavailability of a medical officer to sign the medical certificate also varied. Panglao and Dauis had a reciprocal arrangement between their health centers. In Cortes, the mayor was the alternative authorized person, while in the other municipalities, the reporting waited until the doctor returned.

### Observed Deaths

Combining all sources, 8,075 unique deaths were recorded in the study area between 2002 and 2007, giving an observed crude mortality rate of 7.06 deaths per 1,000 population. Official sources (LCR and HC) recorded 6,696 (83%) of these deaths for a crude mortality rate of 5.85 deaths per 1,000 population. Of the officially recorded deaths (6,696), 6,297 (94%) were recorded by the LCR, and 4,999 (75%) by the HC. The church recorded 6,621 deaths.

Observed LE at birth for the study area (2000-2007) was 67.3 years for males and 74.2 years for females. IMR was 19.2 deaths per 1,000 live births for both males and females, with childhood mortality 23.2 and 23.5 deaths per 1,000 live births for males and females, respectively.

### Two-source capture-recapture analysis

The overall two-source analysis of aggregated official records and church records estimated a total of 8,151 deaths in the study area for 2002-2007. Aggregation of subgroup analyses within strata of age and municipality provided estimates that differed little from the overall estimate. Sensitivity analyses revealed that the source of information on age and municipality (in cases of non-match on these) did not affect results, and the LCR record was used wherever possible to assign values. Official data were found to be virtually independent from church data, with dependence coefficient values greater than 0.99.

**Figure 1:**
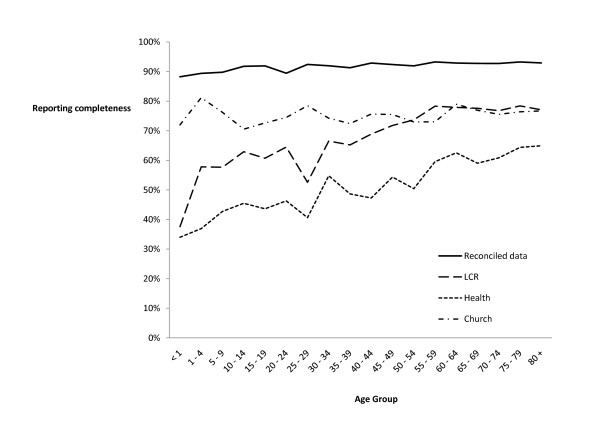
**Proportion of estimated deaths reported, Bohol study area, 2002-2007: two-source analysis**.

Recording completeness varied by age (Figure 1), with child deaths less likely to be recorded than adult deaths. Age had the greatest effect on health center records. Both official sources of data showed increasing completeness with increasing age at death. Variations in reporting by age were not consistent across municipalities. Childhood deaths were less likely to be recorded in Baclayon, Dauis, and Panglao than in the other municipalities.

### Three-source capture-recapture analysis

Estimates of total deaths ranged from 8,137 to 8,983 based on aggregate data, depending on the model selected (Table 1). This equates to deaths from all sources being underregistered by 1% to 10%. Estimates of total deaths varied further when disaggregated by age group and totaled to produce an overall figure.

The saturated model was selected as the best fit (with the lowest significant value for the AIC and BIC) from the analysis of the aggregate data. This also generated the highest estimate of 8,983 total deaths (Table 1). The next best fit falls to any of the models incorporating the association between the LCR and health data.

Pair-wise dependence between sources was, however, not consistent for all age groups. Table 2 shows measures of association between the three sources and confirms the dependence identified in the system review between the LCR and health reporting systems. The odds ratio is high in the dataset as a whole (14.7) and in all three age groups. It is particularly high in persons 5-14 years (12.39) and in persons 15 years and older (15.86). There is additional evidence, however, of an association between the other combinations of sources, although the magnitude of this association as measured by the odds ratio was much smaller (Table 2). This association reaches statistical significance in the dataset as a whole and in persons 15 years and older.

**Table 2:** Association between reporting sources by age-group - three-source analysis, Bohol study area, 2002-2007

Age group	Association	OR	Phi	p
All ages	S1 * S2	14.74 (12.79,17.00)	0.52 (0.01)	< 0.0001
	
	S1 * S3	1.64 (1.30, 2.08)	0.06 (0.02)	< 0.0001
	
	S2 * S3	1.75 (1.53, 1.99)	0.11 (0.01)	< 0.0001

0-4 years	S1 * S2	4.81 (3.26, 7.10)	0.36 (0.04)	< 0.0001
	
	S1 * S3	0.58 (0.31, 1.09)	-0.11 (0.06)	0.0726
	
	S2 * S3	0.85 (0.51, 1.42)	-0.04 (0.06)	0.5373

5-14 years	S1 * S2	12.39 (4.57,33.58)	0.52 (0.08)	< 0.0001
	
	S1 * S3	0.94 (0.17, 5.13)	-0.01 (0.12)	0.9399
	
	S2 * S3	0.98 (0.36, 2.62)	-0.00 (0.10)	0.965

15 + years	S1 * S2	15.86 (13.42, 18.74)	0.50 (0.01)	< 0.0001
	
	S1 * S3	2.01 (1.54, 2.61)	0.08 (0.02)	< 0.0001
	
	S2 * S3	1.78 (1.55, 2.04)	0.11 (0.01)	< 0.0001

Source 1 - LCR data, Source 2 - health data, Source 3 - church data

Taking both the system review and these statistical tests into account, these results did not clearly identify any one model as the only reliable solution, but rather indicated several plausible options depending on the level of age disaggregation used.

### Final mortality estimates

Six models were identified as plausible and a good fit for the data from the two- and three-source analyses. Three yielded low values, and three yielded high values for the total number of deaths (Table 3). These models all accounted for the principal association demonstrated between the LCR and health data, and the differences in reporting by age.

**Table 3:** Final estimates of total deaths by method, Bohol study area, 2002-2007

Method/Model	Total estimated deaths	Crude mortality rate (deaths per 1,000 population)
**Low estimates**		

2-source analysis (combined LCR and health center) including unknown age deaths (with age redistributed by source category) by age	8459 (8226-8692)	7.39 (7.19 - 7.60)

3-source analysis with the 1-2 model using ages redistributed by total age distribution	8457 (8422-8493)	7.39 (7.36 - 7.42)

3-source analysis with the 1-2 model using ages redistributed by total age distribution calculated separately for <5 yrs, 5-14 yrs, and 15+ years)	8481 (8413 - 8546)	7.41 (7.35 - 7.47)

**High estimates**		

3-source analysis (including unknown age deaths without age distribution) - saturated model	8983 (8748-9218)	7.85 (7.64 - 8.06)

3-source analysis with the saturated model using ages redistributed by total age distribution (calculated separately for <5 yrs, 5-14 yrs, and 15+ years)	8964 (8676 - 9305)	7.83 (7.58 - 8.13)

3-source analysis calculated separately for <5 yrs (1-2), 5-14 yrs (1-2), and 15+ years (saturated)	9006 (8734 - 9275)	7.87 (7.63 - 8.11)

**Averages**		

Average of all accepted estimates with maximum 95% confidence intervals (as calculated by any method)	8725 (8354 - 9266)	7.62 (7.19 - 8.13)

The three low estimates were derived from those models that used: a) a two-source analysis (with LCR and health center data combined as a single source against church data) including unknown age deaths (with age redistributed according to source category) by age; b) a three-source analysis with the model accounting for association between the LCR and health data using ages redistributed according to total age distribution; and c) the three-source analysis as defined in (b) with estimates calculated separately for broad age groups (<5 years, 5-14 years, and 15+ years) and summed, rather than as a single application for all ages combined.

The three high estimates were derived from models that used: a) a three-source analysis (including unknown age deaths without age distribution) using the saturated model (model accounting for an association between each possible pair of sources); b) a three-source analysis with the saturated model using ages redistributed according to total age distribution (then calculated separately for <5 years, 5-14 years, and 15+ years); and c) a three-source analysis using ages redistributed according to total age distribution, calculated separately for <5 years (1-2 model), 5-14 years (1-2 model), and 15+ years (saturated model) (Table 3). Low estimates equate to 5% of all deaths in the study area going unreported by any of the sources, while the high estimates set unreported deaths at 10%.

Final estimates were derived by selecting the average of both high and low models and the extreme high and low values of the 95% confidence intervals for any of these models selected. As such, total deaths in the study area for 2002-2007 are estimated to be 8,729, with a range of 8,079 to 9,445 deaths.

**Figure 2:**
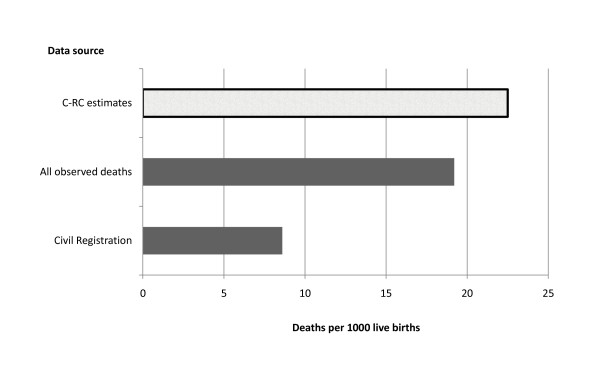
**Estimated infant mortality rate by source, Bohol study area, 2002-2007**.

The final estimates for total deaths imply that for the period 2002-2007, only 77% (95% CI: 71%-83%) of deaths in the study were reported to official sources, with 93% (95% CI: 85%-100%) being reported to any source, including the church. Civil registration was only 72% complete (95% CI: 67%-78%). The crude mortality rate for the study area is therefore estimated at 7.62 deaths per 1,000 population (95% CI: 7.19-8.13). Life expectancy at birth is estimated at 65.7 years for males and 73.0 years for females, one to two years lower than life expectancy estimated from reconciled reported deaths from all sources, and four to five years lower than life expectancy estimated from civil registration data alone. The corrected IMR was 22.7 and 22.2 deaths per 1,000 live births for males and females, respectively, indicating that infant deaths were underreported from all sources by 15% and by 62% from the LCR data (Figure 2). Childhood mortality was 27.3 and 27.1 deaths per 1,000 live births for males and females, respectively, again indicating underreporting by all sources of 13% to 15%. Adult mortality was estimated at 267.2 deaths per 1,000 population for males and 137.3 deaths per 1,000 population for females, indicating underreporting by the LCR of 24% to 29%.

## Discussion

These findings demonstrate that reporting of deaths in Bohol to both official and church sources is incomplete. Estimates suggest that only 77% of deaths are recorded in official LCR or health records, while 5% to 10% of all deaths are not reported to any source. Civil registration was estimated to be only 72% complete for all ages. Although the impact of underreporting of deaths on summary mortality measures such as LE at birth was in the order of four to five years, there was a much greater impact on age-specific measures of IMR and childhood mortality, which were 15% to 18% higher than those calculated from the unadjusted reconciled data. Infant deaths were underreported by 62% in the civil registration data. Adult mortality was also 11% higher than the original estimate from the reconciled data.

The three-source analysis allows for departure from the assumption of independence between sources, essential for the two-source analysis. However, the addition of any further source to the capture-recapture analysis increases the uncertainty in the estimate and the width of confidence intervals [17,19]. The additional models generated for each possible set of associations between sources as additional sources are added [17] results in a greater onus on researchers to select an appropriate model based on their knowledge of the reporting systems. Conducting the three-source analysis is therefore only justified if the third source contributes deaths not recorded by the other two sources to the analysis. The two-source analysis found very weak association between official and church records. Although the three-source analysis confirmed this, the association was statistically significant and thus should not be ignored (Table 2). The two-source analysis is viewed as a lower limit for the estimate of total deaths, as the association indicates that deaths reported to official sources are more likely to also be reported to the church, resulting in an underestimation of unreported deaths. The three-source results accounting for this dependence therefore range upward from the two-source estimate. Since one model cannot be identified as the only model to fit the data, the various models are used to set upper and lower limits of the estimate of unreported deaths, and therefore total deaths, for the study area.

Population estimates were derived from the 2000 [14] and 2007 [15] censuses, using linear interpolation to provide estimates for intermediate years, and are thus more likely to have overestimated the population for intermediate years, biasing mortality rates downward. The broad matching criteria used to reconcile the data sources and multiple checks of the matching process minimize the risk of significant errors in the reconciliation of data sources. Further, in this setting, undermatching due to incomplete data is more likely than overmatching. This would lead to overestimation of the number of deaths not reported [17], and therefore lead to an inflated estimate of mortality, thus supporting the use of the higher estimates of total deaths as an upper limit.

The small number of deaths in the study area meant that while disaggregation by 5-year age group or municipality was possible with the two-source analysis, many of the results became unstable when the data were either disaggregated further by year, or when the official sources were separated for the three-source analysis. The two-source analysis was therefore used to assess variations in reporting patterns. The important difference in reporting completeness between childhood deaths and adult deaths was accounted for in the three-source analysis by disaggregating data by broader age groups (0-4, 5-14, and 15+ years).

Capture-recapture analysis requires that: the record sources capture deaths from the same population, the population is closed, and that every death has the same probability of being reported [17,18]. The analysis is also affected by the dependence between the data sources, with independence required for an accurate result from a two-source analysis. These conditions, however, are rarely fully met with human populations, and such analysis therefore requires a solid understanding of reporting systems in order to assess the magnitude of any dependence and population movements in or out of the area. In the Bohol study area, while the population is not strictly closed, migration both in and out is low, with the latest census reporting an annual growth rate of 1.06 percent for the period 2000 to 2007, although most of this change is accounted for through births and deaths [3]. Dependence between the LCR and health reporting systems was accounted for in both the two- and three-source analyses, and the high proportion of Catholics in the study population meant that for nearly all deaths, there was a reasonable potential for that death to be reported to the local parish and thus be included in this study. As such, while there were minor deviations from the assumed conditions for a capture-recapture analysis, Bohol is an ideal situation in which to conduct this analysis, and results are highly unlikely to have been affected by assumptions of the method being violated. The selection of municipalities based on proximity to the urban center of Tagbilaran would likely result in the estimates of completeness presented here being considered a best-case scenario, with reporting from outlying areas likely to be even less complete. Additionally, the gender and age distribution in these areas is almost identical to that for the broader province [14,15], suggesting that there is some generalizability to the local area. However, the variation in reporting patterns and procedures by municipality identified by this study, despite national legislation and procedural policy, indicates that it is not appropriate to apply these findings and assessment to other areas of the Philippines without an understanding of reporting processes at a local level.

While there have been very few studies on reporting completeness in the Philippines, our findings of reporting completeness to official sources are consistent with a 2003 evaluation of mortality data in the Philippines based on data obtained by the World Health Organization [2]. Completeness of death registration is often assessed as part of the analyses of census data; however, this has not been reported in either of the two most recent censuses, 2000 and 2007, in the Philippines [14,15]. Although estimates for this study were calculated from only six of the 48 municipalities in Bohol, they are significantly higher than official published estimates that put IMR for Bohol province at 14.6 in 2000 and 8.53 in 2004 [4]. Published estimates of LE at birth for Bohol vary, with estimates for 2000-2005 from official sources in the Philippines ranging from 65.3 to 68.19 for males and 71.0 to 72.9 for females (2000-2005) [3,4]. LE estimates from this study are consistent with the lower published estimates from the National Statistics Office (NSO) for males, but are higher than the published estimates for females [3]. As both sets of published data are derived from civil registration within the province, it is probable that estimates released by the NSO have been corrected for some undercounting in the data.

## Conclusions

This study demonstrates that even though the Philippines has national legislation and policies on death registration, there is significant underreporting of deaths to official sources in Bohol. While this had only a small impact on summary measures of mortality, such as the crude death rate and LE, the impact of underreporting on age-specific measures such as IMR and childhood mortality was much greater. Uncorrected mortality measures would subsequently be misleading if used for health planning and evaluation purposes. These findings highlight the importance of ensuring that official mortality estimates from the Philippines are derived from data that have been assessed for underreporting and corrected if necessary.

Official LCR and health sources were more incomplete that church records across nearly all age groups (Figure 1), highlighting the potential to improve reporting to official sources by developing stronger links between local civil registrars and parish churches in this setting. Completeness of death reporting within the health system could be substantially improved by reducing losses from the system through ensuring that a copy of each medical certificate of death is either kept or entered into a logbook rather than relying on the data being returned from the LCR. Variations in local reporting practices further indicate that additional improvements in reporting completeness may be possible through adapting local practices to align with a standard national approach.

Finally, capture-recapture methods are an important tool for assessing and correcting mortality data for reporting completeness, and have been demonstrated here to provide improved understanding of mortality estimates derived from a system generally recognized to be of reasonable quality. While not an entirely new application of capture-recapture analysis, the method has potential to significantly improve our understanding of mortality in settings where multiple incomplete datasets are available, particularly in small areas (such as the small countries of the Pacific Islands or provinces of the larger countries of Asia) where it is possible for researchers to develop a strong understanding of system processes in order to make sensible decisions about both model selection and matching criteria.

## Competing interests

The authors declare that they have no competing interests.

## Authors' contributions

KC designed the study; conceived and conducted the system review; designed and tested matching criteria; prepared the data for the three-source analysis; performed the statistical analysis for the two-source analysis and final model selection including interpretation of final results; and drafted the manuscript. GW made substantial contributions to analysis and interpretation of data, performed the statistical analysis for the three-source analysis and revised the manuscript critically. VT coordinated data collection, collation and matching, and made substantial contributions to the interpretation of data. DS coordinated initial data cleaning, management, and tabulation of records, and made substantial contributions to the interpretation of data.

HM made substantial contributions to the acquisition and interpretation of data. IR conceived and coordinated the study and revised the manuscript critically for important intellectual content. All authors read and approved the final manuscript.
